# Optical decomposition of DNA gel and modification of object mobility on micrometre scale

**DOI:** 10.1038/s41598-019-56501-z

**Published:** 2019-12-27

**Authors:** Suguru Shimomura, Takahiro Nishimura, Yusuke Ogura, Jun Tanida

**Affiliations:** 1 Department of Information and Physical Sciences, Graduate School of Information Science and Technology, Osaka University, 1-5 Yamadaoka, Suita, Osaka, 565-0871 Japan; 2 Division of Sustainable Energy and Environmental Engineering, Graduate School of Engineering, Osaka University, 2-1 Yamadaoka, Suita, Osaka, 565-0871 Japan

**Keywords:** Biomedical engineering, Biophotonics

## Abstract

DNA gels can be engineered to exhibit specific properties through the choice of DNA sequences and modification with dye molecules, and can therefore be useful in biomedical applications such as the detection of biomolecules. State transitions of DNA gels on the micrometre scale can generate a viscosity gradient, which can be used to modify the mobility of micrometre-sized objects. In this paper, we propose a method for changing the viscosity of DNA gels using optical decomposition. The use of light allows for decomposition on the micrometre scale, which can be used to achieve patterned viscosity changes within DNA gels. Decomposition was induced by thermal energy released through non-radiative relaxation of excited quenchers. We demonstrated the decomposition of DNA gels in response to irradiation patterns on the micrometre scale. In addition, as a result of changes in DNA gel viscosity due to decomposition, the mobility of polystyrene beads was shown to increase. This technique could provide a new optical approach for controlling the mobility of micrometre-sized objects.

## Introduction

The use of purpose designed DNA sequences can allow for the self-assembly of various structures from the nanometre to millimetre scale^1–5^. DNA gels, which are formed by crosslinking numerous DNA motifs^6–8^, can transition to the sol state in response to temperature^9^, pH^10^ or the presence of specific molecules^11^, and are biocompatible and useful in bioesensing. For example, the state change of DNA gels can be applied to the detection of biomolecules such as cocaine^12^ and thrombin^13^. In addition, live cells can be trapped selectively by cloaking circulating tumour cells with DNA gels in response to the presence of epithelial cell adhesion molecules^14^. This suggests that DNA gels have significant potential to regulate the mobility of micrometre-scale objects, including cells. However, these functions are based on a passive reaction, and the mobility cannot be modulated dynamically. The control of an object’s motion by changing the viscosity of DNA gels would provide a method for the sorting and arrangement of cells, which are necessary steps in the creation of cellular tissues^15^. To realise this, patterning by spatially controlling the state transition of DNA gels is required. Previous research in DNA gel patterning includes an ink-jet method using different DNA solutions^16^ and a patterning method using a substrate coated with DNA initiators^17^. However, scanning nozzles containing DNA solutions or coating DNA strands on a substrate can result in slow and complicated DNA gel production.

Optical methods provide a promising solution to this problem owing to their features, which include spatial parallelism and controllability. Light irradiation can remotely induce a gel-sol transition^18–20^, swelling, and shrinking^21^. Moreover, light patterns can be generated and dynamically changed using a spatial light modulator (SLM), which makes rapid processing at the micrometre scale possible. We have already demonstrated optical fabrication of DNA gels in a few seconds by using a photothermal conversion effect of dark quenchers^22^. This method enables us to control the amount of DNA gel produced and any gel patterning on the basis of the light power distribution on the micrometre scale, which initiates a viscosity increase and leads to reduced mobility. The reverse method of DNA gel decomposition provides a different mode of mobility modulation. The decomposition of DNA gels leads to a reduction of their viscosity and an increase in the mobility of associated objects. For flexible control of object mobility, it is important to realise the spatial decomposition of DNA gels.

In this paper, we propose a method for controlling the mobility of micrometre-sized objects through optical decomposition of DNA gels. The use of light enables the decomposition of the DNA gels on a micrometre scale. Moreover, the local reduction of DNA gel viscosity through irradiation enhances the motion of associated objects. To demonstrate the spatial and temporal shaping of DNA gels, we acquired fluorescence images of DNA gels stained with fluorescent dyes with various irradiation patterns. In addition, the degree of motion of micrometre-sized beads in response to irradiation was measured to verify the modulation of object mobility as a result of decomposition.

## Results

### Optical decomposition of DNA gels

**Figure 1:**
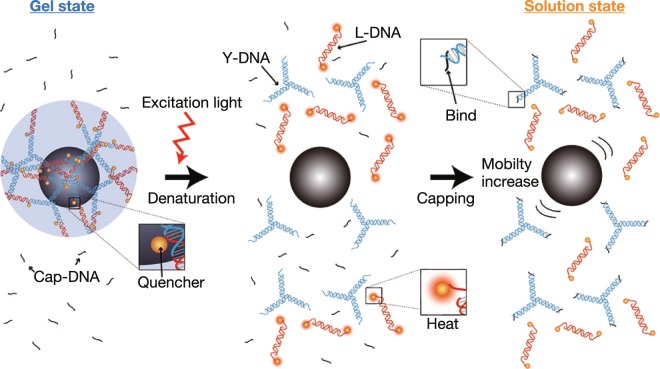
Schematic diagram of DNA gel decomposition as a result of light irradiation.

A schematic diagram of mobility modification as a result of optical decomposition of DNA gels is shown in Fig. 1. The sequences of the individual DNA components are noted in the Materials and Methods section. For optical decomposition, the sticky ends of the L-DNA were modified with quenchers. In the initial state, the DNA gels encapsulate micrometre-sized objects and regulate their mobility. Optical excitation causes quenchers to generate thermal energy through a non-radiative relaxation process^23^. The energy dissipated from excited quenchers is sufficient to denature 10-bp double-stranded DNA (dsDNA) to two single DNA strands (ssDNAs)^24^. Thus, the bonds between the Y-DNA and the L-DNA in the DNA gel are cleaved by light excitation, and the DNA gels within the irradiation area are decomposed. The viscosity then decreases owing to the state change of the DNA gel, and the mobility of the micrometre-sized objects is enhanced. After the irradiation, the cleaved sticky ends of Y-DNA bind to ssDNA (Cap-DNA) that prevents rebinding with L-DNA due to the presence of a large amount of Cap-DNA. As a result, the DNA solution maintains its state and viscosity even after the irradiation is stopped. The decomposition ratio depends on the light intensity; therefore the mobility of each object can be modulated according to a light pattern.

**Figure 2:**
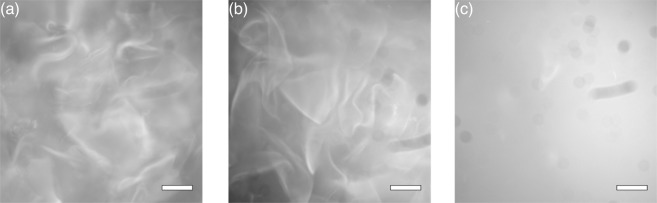
Fluorescence images of a DNA gel containing all of the DNA components (**a**) in the initial state, (**b**) after 1 h without irradiation, and (**c**) after 1 h with irradiation. Scale bars indicate 200 *μ*m.

First, we created DNA gels in a DNA solution containing all of the DNA components and observed it using a fluorescence microscope. To identify the DNA gel, the DNA solution was stained with a fluorescent dye, DAPI (Dojindo Molecular Technologies, Inc., absorption wavelength: 360 nm, fluorescence wavelength: 460 nm), which selectively binds to dsDNAs. Since DNA gels are constructed from many DNA motifs, their DNA density and the fluorescence that indicates the existence of Y-DNAs and L-DNAs are higher than those in the DNA solution. Figure 2(a) shows a fluorescence image of the DNA solution. A high fluorescence-intensity area was observed in the DNA solution, which shows that DNA gels were formed as anticipated. Next, we investigated the decomposition of DNA gels by light irradiation. Two test tubes of DNA solution containing the same components as in the first experiment were prepared. One test tube was not irradiated, and the other was exposed to excitation light (wavelength: 660 nm, intensity: $3.0\times {10}^{-1}\,{\rm{W}}/{{\rm{cm}}}^{2}$) emitted from a laser diode (ML101J27, Mitsubishi Electric) for 1 h. Figure 2(b,c) show fluorescence images of the non-irradiated and irradiated samples, which were dropped onto glass slides, respectively. The variation of fluorescence intensity in the irradiated sample is much less than that in the non-irradiated sample. These results indicate that the DNA gels were decomposed by light irradiation.

### Patterning of DNA gels using light patterns

**Figure 3:**
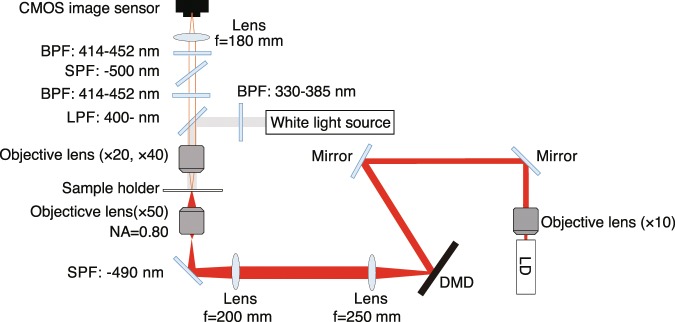
Optical setup for DNA gel patterning. BPF: band pass filter, SPF: short pass filter, LD: laser diode f: focal length.

**Figure 4:**
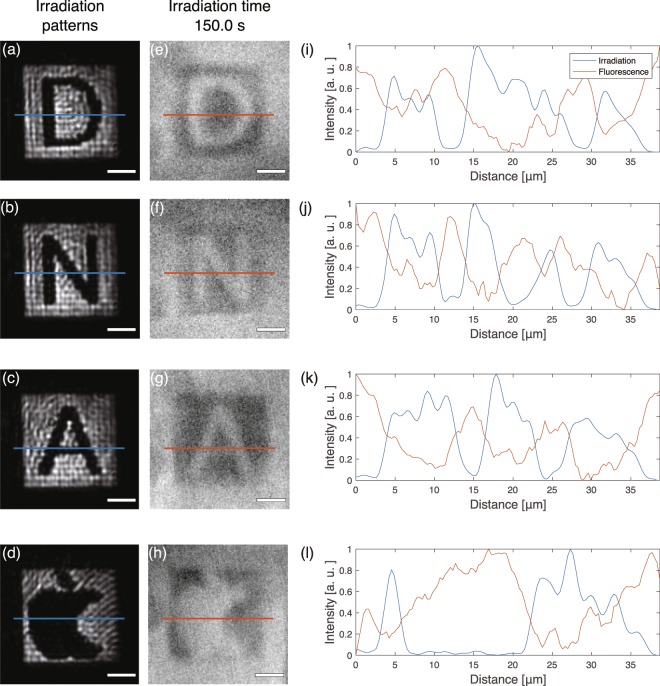
Irradiation patterns for (**a**) D, (**b**) N, (**c**) A, and (**d**) a bitten apple. (**e**–**h**) Fluorescence images obtained under irradiation at 150.0 s. (**i**–**l**) Intensity profiles along the blue and red lines shown in (**a**–**h**), respectively. Scale bars indicate 10 *μ*m.

To demonstrate the spatial decomposition of DNA gels, we irradiated the DNA solution with several optical patterns and captured fluorescence images. Figure 3 shows the optical setup used to pattern DNA gels. A beam emitted from a laser diode (wavelength: 660 nm) was guided to a digital mirror device (DMD) following reflection by two mirrors. The beam was modulated by the DMD, and the optical pattern just after the DMD was imaged at the sample plane through imaging optics including an objective lens. Figure 4(a–h) show the optical patterns used in the experiment and fluorescence images of the individual irradiation patterns, respectively. The fluorescence intensity within the area of high irradiation intensity began to decrease and became equivalent to that outside the pattern at 150.0 s (see Movies 1–4). Figure 4(i–l) show the profiles taken along the blue and red lines in Fig. 4(a–h), respectively. In the area of high light intensity, the fluorescence intensity was low. This result shows that the DNA gels were decomposed according to the irradiation patterns used. Interestingly, the fluorescence increased in the irradiation area at the beginning of the irradiation and then decreased. This fluorescence persisted even when the irradiation was stopped. A possible reason for this could be that quenchers on the L-DNAs went away from the DNA gels, and more of the excitation energy of the DAPI was converted to fluorescence. The quenchers on the L-DNAs can absorb the energy of the DAPI when the quenchers are close to the DAPI^25^. In the initial state, the DAPI in the DNA gels not only emitted fluorescence but also transferred the excitation energy to the quenchers at the same time. When the DNA gels began to be decomposed, the L-DNAs were cleaved from the DNA gels, and fluorescence of the DAPI in DNA gels increased due to the absence of quenchers. Actually, the fluorescence intensity of the DNA solution was higher when the Y-DNAs binded with Cap-DNAs compared with when they binded with L-DNAs (see Materials and Methods). After sufficient irradiation time, the DNA gels were decomposed because the sticky ends of the Y-DNAs binded to Cap-DNA, and the DNAs in the irradiation area were simultaneously dispersed by the thermal gradient generated by the excited quenchers. Therefore, suitable irradiation is required for the decomposition and resulting decrease in viscosity.

### Modification of polystyrene bead mobility by optical decomposition of DNA gel

**Figure 5:**
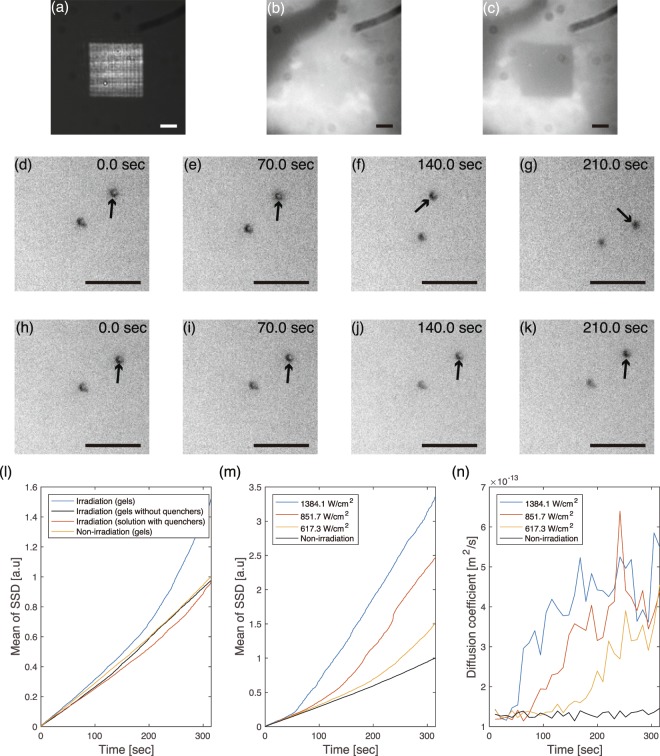
(**a**) Irradiation pattern. Fluorescence images (**b**) before and (**c**) after irradiation for 310.0 s. Time course of the bead’s movements (**d**–**e**) with irradiation and (**h**–**k**) without irradiation. The same PSB at each frame is indicated by black arrows. Scale bars indicate 10 *μ*m. (**l**,**m**) The relationship between time and total square displacement of the PSBs. (**n**) Diffusion coefficient over time.

The decrease in viscosity caused by spatially controlled optical decomposition of DNA gels can be applied to modify the mobility of micrometre-sized objects. To confirm this, we measured the Brownian motion of polystyrene beads (PSBs) encapsulated in the DNA gels. For the decomposition, a square pattern (intensity: 617.3 W/cm^2^) shown in Fig. 5(a) was used to irradiate the DNA gels containing PSBs (Polysicences, Inc., 07310, diameter: 1.0 *μ*m), and we captured bright-field images of the PSBs every 0.35 s. The decomposition of the DNA gels was confirmed from the fluorescence images before and after irradiation (Fig. 5(b,c)). The bright-field images of the PSBs in DNA gels under irradiation and under non-irradiation at each time (0.0, 70.0, 140.0, and 210.0 s) are shown in Fig. 5(d–k), respectively. The PSBs under irradiation gradually moved while the positions of the PSBs under non-irradiation hardly changed (the difference of the movement is also shown in Movie 5). We also measured the displacement distances of five PSBs between each frame, and calculated the mean and variance of the sum of squared displacements (SSD, *r*_*t*_) at time *t* (see Materials and Methods section for the definition). Figure 5(l) shows the relationship between the mean SSDs of the PSBs and observation time. Each SSD was normalised by using the maximum value of SSDs measured for the sample of the same composition under the non-irradiation condition. Without irradiation, the SSDs increased linearly owing to Brownian motion. In contrast, the displacement under irradiation was higher than that in the non-irradiation case, and the rate of increase of the SSD under irradiation became greater as time progressed. The normalised SSDs of the PSBs in the DNA gels without the quenchers and in the DNA solution including L-DNAs did not nonlinearly increase even under irradiation. These results show that the degree of Brownian motion increased due to the reduced DNA gel viscosity by thermal energy not of irradiation light itself but of excited quenchers. To verify that the viscosity was modulated according to irradiation conditions, we measured the SSD under light irradiation with various intensities. Figure 5(m) shows the mean of SSDs under the irradiation condition with light intensities of 1384.1, 851.7, and 617.3 W/cm^2^. The degree of motion of the PSBs increased with increasing light intensity (see Movie 6). Furthermore, the dependence of the diffusion coefficient obtained from these measurements on the time is shown in Fig. 5(n). The diffusion coefficient rapidly increased after 31.5, 63.0, and 136.5 s, respectively. These results revealed that the DNA gels began to decompose after those times. Dynamic viscosity is inversely proportional to the diffusion coefficient^26^, and the viscosity values before and after irradiation with 617.3 W/cm^2^ were estimated to be 3.0 and 1.0 mPa · s. Therefore, the viscosity decrease can be controlled by adjusting the irradiation conditions, and the mobility of PSBs, which depends on the viscosity, can be temporally modulated by light irradiation. It is expected that objects could be guided to given positions by controlling the light pattern design.

## Conclusion

We have presented a method for modifying the mobility of a micrometre-sized object by optical decomposition of a DNA gel. Thermal energy from optically excited quenchers in the irradiation area induced the decomposition of DNA gels. We demonstrated the pattering of DNA gels on a micrometre scale using light pattern irradiation. Furthermore, the viscosity decrease due to the optical decomposition of the DNA gels increased the mobility of the PSBs. Spatial viscosity control of DNA gels will enable remote modulation of the motion of micrometre-sized objects using suitable irradiation patterns. Our method provides a new approach for controlling the motion of micrometre-sized objects. It is expected that combining methods for fabricating DNA gels with those for controlling their viscosity will allow the construction of complex cell tissue and analysis of cell motion.

## Materials and Methods

### DNA sequences used to construct the DNA gels and the sample preparation procedure

The designed DNA components are listed in Table 1. All DNA strands were purchased from TsukubaOligo Service Co. Ltd. The Y-DNA and the L-DNA consisted of three (Y1, Y2, Y3) and two (L1, L2) kinds of DNA strands, respectively. The 5′- end of L1 and of L2 was modified with BlackBerry Quencher 650 (BBQ-650), which is a quencher absorbing light in the wavelength range 550–750 nm. DNA gels were created with these DNA components (with concentrations Y-DNA: 28.6 *μ*M, L-DNA: 28.6 *μ*M, and Cap-DNA: 228.8 *μ*M) in buffer (NaCl: 7.1 mM, H_3_PO_4_: 2.2 mM). To create sufficient DNA gels, Y-DNAs and L-DNAs were mixed in two test tubes and the temperature was maintained at 6 °C. After 3 h, Cap-DNAs were added to both samples. Each sample was dropped onto a glass slide and was observed using a fluorescence microscope (BX51WI, Olympus Corp.). To capture the images in Fig. 2, we used a complementary metal-oxide-semiconductor (CMOS) image sensor (C13440-20CU, Hamamatsu Photonics K.K., pixel pitch: 6.5 × 6.5 *μ*m^2^), an objective lens (UMPlan FI, Olympus Corp., ×10), a dichroic mirror (transmission wavelength: 400 nm-), and two band-pass filters (transmission wavelengths: 330–385 nm for excitation, and 414–452 nm for detection). The fluorescence excitation light source was a halogen lamp.

**Table 1:** DNA sequences and modifications.

Strand name	Sequence
Y1	5′-CGATTGACCACGCTGTCCTAACCATGACCGTCGAAG-3′
Y2	5′-CGATTGACCTTCGACGGTCATGTACTAGATCAGAGG-3′
Y3	5′-CGATTGACCCTCTGATCTAGTAGTTAGGACAGCGTG-3′
L1	5′-(BBQ-650)-GTCAATCGTCTATTCGCATGAGGATCCCATTCACCGTAAG-3′
L2	5′-(BBQ-650)-GTCAATCGCTTACGGTGAATGGGATCCTCATGCGAATAGA-3′
Cap-DNA	5′-GTCAATCG-3′

### Light pattern irradiation conditions and adjustment of figures and line profiles

The optical setup shown in Fig. 3 was constructed by combining with the fluorescence microscope (BX51WI, Olympus Corp.). For observation of the fluorescence of DNA gels, the irradiation light was cut off by using two bandpass filters (transmission wavelength: 414–452 nm), and a shortpass filter (transmission wavelength: 500 nm). The intensity of each light pattern in Fig. 4(a–d) was (a) 570.0, (b) 558.9, (c) 553.0, and (d) 469.2 W/cm^2^ on the sample plane. These densities are values averaged over the pattern area. To generate the light patterns, a DMD (Discovery 1100, Texas Instruments, pixel pitch: 13.68 *μ*m) was used. The images of the light patterns and the corresponding fluorescence images were captured using objective lenses with magnifications of 40× (156403, Olympus Corp.) and 20× (LWD MSPlan20, Olympus Corp.), respectively. The exposure time was 0.5 s, so that the frame rate for capturing the fluorescence images was 2.0 fps. In this experiment, a sample holder was made from a cover slip, a glass slide, and double-sided tape. The sample was placed in the region surrounded by the double-sided tape and sandwiched between the cover slip and the glass slide. The contrast of each image was adjusted so that the minimum and maximum pixel values of each image were 0 and 255, respectively. The line profiles shown in Fig. 4(i–l) show data sequences smoothed by using a five-point moving average filter, and these intensities were normalised so that the minimum and maximum intensities in each line were 0 and 1.

### Fluorescence intensity of DNA solution depending on the binding state of DNA components

**Figure 6:**
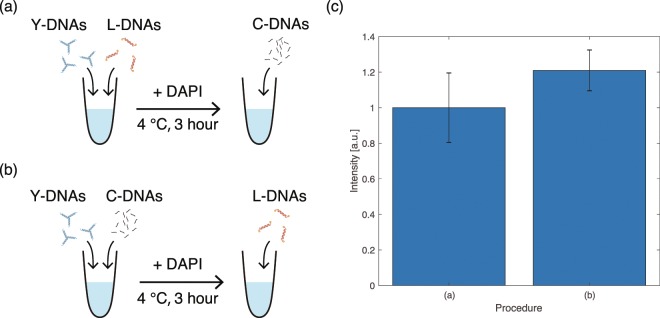
Sample preparation procedures of DNA solution in which the Y-DNAs bind to (**a**) the L-DNAs and (**b**) to the Cap-DNAs. (**c**) Normalised fluorescence intensity of the samples prepared by methods (**a** and **b**).

To investigate whether the intensity of DAPI increases when Y-DNAs are separated from L-DNAs and bind with Cap-DNAs, we measured the fluorescence intensity of two DNA solutions at different states using a fluorescence spectrophotometer (JASSO corp., FP-6200). We prepared two kinds of samples with different procedures shown in Fig. 6(a,b). The first sample contained Y-DNAs and L-DNAs, the other contained Y-DNAs and Cap-DNAs. After mixing DAPI dyes with each of the samples and keeping the temperature at 6 °C for 3 hours, Cap-DNAs and L-DNAs were added to the individual samples. While Y-DNAs bind with L-DNAs in the DNA solution shown in Fig. 6(a), they bind with Cap-DNAs in the DNA solution shown in Fig. 6(b) because Cap-DNAs prevent Y-DNAs from binding with L-DNAs. Figure 6(c) shows the fluorescence intensity of the DNA solutions measured immediately after the sample preparation. The irradiation wavelength for excitation of DAPI was 360 nm, and the detected fluorescence wavelength was 460 nm. We measured six samples prepared by each procedure, and the intensity values were normalised by the mean value for the DNA solution prepared shown in Fig. 6(a). The fluorescence intensity of the DNA solution containing Y-DNAs binding with L-DNAs was lower than that containing Y-DNAs binding with Cap-DNAs. These results show that fluorescence intensity increases when Y-DNAs in the DNA gels are cleaved from L-DNAs and bind with Cap-DNAs.

### Motion analysis of SSD and PSB observation environment in DNA gels

To evaluate the degree of motion, SSD *r*_*t*_ at observation time *t* is defined as

1 $${r}_{t}=\mathop{\sum }\limits_{i=1}^{t}\,{(x({t}_{i})-x({t}_{i-1}))}^{2},$$

where *t*_*i*_ is the time for the *i*-th frame in a sequence of images, and $x({t}_{i})$ is the position at *t*_*i*_. To detect the position of a PSB from individual images, we used the *imfindcircles* function of MATLAB and obtained the center of the PSB detected by Hough transformation of the images. The individual images shown in Fig. 5 were captured by using the optical setup shown in Fig. 3. The DNA gels containing PSBs were created by mixing the Y-DNA solution with the PSBs and the L-DNA solution. After 3 h and whilst maintaining the temperature of the DNA solution at 6 °C, the samples were placed on the sample holder and observed at room temperature. Instead of the double-sided tape in the sample holder used in the patterning experiment, we used additional PSBs with a different size (Polysciences, Inc., 17136-5, diameter: 10.0 *μ*m) to create the gap between the glass slide and the cover slip. This gap limited the movement of the PSBs within the observable depth, and thus the position could be measured. The diffusion coefficients in DNA gels under non-irradiated and irradiated conditions were measured from the mean square displacement of beads over 10.5 s. The dynamic viscosities before and after irradiation were obtained from the values of the diffusion coefficients at 10.5 s and 310.0 s. Each value was estimated using the Stokes-Einstein equation^26^. In the calculation, the temperature of the DNA solution was assumed to be room temperature, 23 °C.

## Supplementary information

Movie 1

Movie 2

Movie 3

Movie 4

Movie 5

Movie 6

## References

[1] Seeman NC (1982). Nucleic acid junctions and lattices. Journal of Theoretical Biology.

[2] Winfree E, Liu F, Wenzler LA, Seeman NC (1998). Design and self-assembly of two-dimensional DNA crystals. Nature.

[3] Rothemund Paul W. K. (2006). Folding DNA to create nanoscale shapes and patterns. Nature.

[4] Zheng J (2009). From molecular to macroscopic via the rational design of a self-assembled 3D DNA crystal. Nature.

[5] Tikhomirov Grigory, Petersen Philip, Qian Lulu (2017). Fractal assembly of micrometre-scale DNA origami arrays with arbitrary patterns. Nature.

[6] Li Y (2004). Controlled assembly of dendrimer-like DNA. Nature materials.

[7] Um SH (2006). Enzyme-catalysed assembly of DNA hydrogel. Nature materials.

[8] Lee JB (2012). A mechanical metamaterial made from a DNA hydrogel. Nature nanotechnology.

[9] Xing Y (2011). Self-assembled DNA hydrogels with designable thermal and enzymatic responsiveness. Advanced Materials.

[10] Cheng E (2009). A pH-triggered, fast-responding DNA hydrogel. Angewandte Chemie - International Edition.

[11] Guo W (2014). Reversible Ag(+)-crosslinked DNA hydrogels. Chemical communications (Cambridge, England).

[12] Zhu Z (2010). An aptamer cross-linked hydrogel as a colorimetric platform for visual detection. Angewandte Chemie - International Edition.

[13] Zhang L, Lei J, Liu L, Li C, Ju H (2013). Self-assembled DNA hydrogel as switchable material for aptamer-based fluorescent detection of protein. Analytical Chemistry.

[14] Song P (2017). DNA Hydrogel with Aptamer-Toehold-Based Recognition, Cloaking, and Decloaking of Circulating Tumor Cells for Live Cell Analysis. Nano Letters.

[15] Jin J (2013). A triggered dna hydrogel cover to envelop and release single cells. Advanced Materials.

[16] Li C (2015). Rapid formation of a supramolecular polypeptide-DNA Hydrogel for *in situ* three-dimensional multilayer bioprinting. Angewandte Chemie - International Edition.

[17] Wang J (2017). Clamped Hybridization Chain Reactions for the Self-Assembly of Patterned DNA Hydrogels. Angewandte Chemie - International Edition.

[18] Lee JI, Kim HS, Yoo HS (2009). DNA nanogels composed of chitosan and Pluronic with thermo-sensitive and photo-crosslinking properties. International Journal of Pharmaceutics.

[19] Kang Huaizhi, Liu Haipeng, Zhang Xiaoling, Yan Jilin, Zhu Zhi, Peng Lu, Yang Huanghao, Kim Youngmi, Tan Weihong (2011). Photoresponsive DNA-Cross-Linked Hydrogels for Controllable Release and Cancer Therapy. Langmuir.

[20] Kandatsu D (2016). Reversible Gel - Sol Transition of a Photo-Responsive DNA Gel. ChemBioChem.

[21] Cangialosi A (2017). DNA sequence-directed shape change of photopatterned hydrogels via high-degree swelling. Science.

[22] Shimomura Suguru, Nishimura Takahiro, Ogura Yusuke, Tanida Jun (2018). Photothermal fabrication of microscale patterned DNA hydrogels. Royal Society Open Science.

[23] Gaiduk A., Yorulmaz M., Ruijgrok P. V., Orrit M. (2010). Room-Temperature Detection of a Single Molecule's Absorption by Photothermal Contrast. Science.

[24] Ogura Y, Onishi A, Nishimura T, Tanida J (2016). Optically controlled release of DNA based on nonradiative relaxation process of quenchers. Biomedical Optics Express.

[25] Marras SA, Kramer FR, Tyagi S (2002). Efficiencies of fluorescence resonance energy transfer and contact-mediated quenching in oligonucleotide probes. Nucleic acids research.

[26] Edward JT (1970). Molecular volumes and the stokes-einstein equation. Journal of Chemical Education.

